# Humanized microbiota mice as a model of recurrent *Clostridium difficile* disease

**DOI:** 10.1186/s40168-015-0097-2

**Published:** 2015-08-20

**Authors:** James Collins, Jennifer M. Auchtung, Laura Schaefer, Kathryn A. Eaton, Robert A. Britton

**Affiliations:** Baylor College of Medicine, Department of Molecular Virology and Microbiology, Alkek Center for Metagenomics and Microbiome Research, Houston, TX USA; Department of Microbiology and Immunology, University of Michigan, Ann Arbor, MI USA

**Keywords:** *C. difficile*, Microbiota, Mouse model

## Abstract

**Background:**

*Clostridium difficile* disease is the leading antibiotic-associated cause of diarrhea and nosocomial acquired infection in the western world. The per annum burden in the USA alone amounts to 250,000 cases with 14,000 ascribed deaths and medical costs in excess of a billion dollars. Novel models for the study of *C. difficile* infection are therefore pertinent.

**Results:**

Germ free C57BL/6 mice gavaged with a healthy human fecal microbiota maintained a stable “humanized” microbiota over multiple generations when housed under specific pathogen-free (SPF) conditions. As with mice containing a conventional microbiota, treatment with a five-antibiotic cocktail followed by a single dose of clindamycin renders the animals susceptible to *C. difficile* infection (CDI). Interestingly, after recovery from the initial CDI infection, a single intraperitoneal injection of clindamycin is sufficient to induce CDI relapse. Relapse of CDI can be induced up to 35 days postinfection after recovery from the initial infection, and multiple episodes of relapse can be induced.

**Conclusions:**

This model enables the study of recurrent *C. difficile* disease in a host containing a human-derived microbiota. Probiotic treatments using human-derived microbes, either prophylactic or curative, can be tested within the model. The identification and testing of human-derived microbial communities within a humanized microbiota mouse model may enable a higher rate of successful transfer of bacteria-based treatments from the lab to human patients due to the microbes involved initiating from, and being adapted to, the human GI tract.

**Electronic supplementary material:**

The online version of this article (doi:10.1186/s40168-015-0097-2) contains supplementary material, which is available to authorized users.

## Background

*Clostridium difficile* infection (CDI) is the principle cause of antibiotic-associated diarrhea (AAD) and nosocomial infection in the western world. Severe cases lead to pseudomembranous colitis and toxic megacolon which can require drastic surgical intervention. Recurrence occurs in approximately 25 % of cases treated with metronidazole or vancomycin with 12 % percent of patients experiencing at least two recurrences and 6 % an excess of two [1]. The per annum burden in the USA amounts to 250,000 cases with 14,000 ascribed deaths and medical costs in excess of a billion dollars [2].

Disruption of the host microbiota, whose functions include the prevention and limitation of pathogen colonization and growth, is the most prominent risk factor in CDI [3]. Recently, the recapitulation of this barrier, via fecal microbial transplantation (FMT), has shown great promise as a treatment for recurrent CDI cases, recalcitrant to other treatments [4]. FMT, however, has not yet been approved by the FDA and there are safety concerns over the use of a complex mix of bacterial, viral, and fungal components in addition to prions and potentially unknown biologically active substances [5].

Numerous animal [6] and in vitro [7, 8] models have been used to study *C. difficile* biology, virulence factors, and associated disease. Until recently, the golden Syrian hamster was the most widely used model of CDI. However, hamsters rapidly succumb to disease, often becoming moribund within 48 h, and are not suitable for studies of recurrent disease. Therefore, researchers have shifted towards the use of mouse models of CDI, and a few different models for the study of *C. difficile* relapse/recurrence have been developed. These models rely upon repeat antibiotic treatment with or without re-infection [9, 10] or suppression of initial infection with antibiotics [11] to achieve a relapse-like state. To our knowledge, animal models to study CDI relapse/recurrence in the presence of a human-derived microbiota have not been described. Here, we characterize a humanized microbiota mouse (^HMb^mouse) model of CDI with the ability to induce relapse/recurrence of disease in otherwise healthy mice by the administration of a single dose of clindamycin. Investigation of the changes in microbiota that lead to susceptibility to CDI may facilitate a targeted approach to identifying therapeutic microbes. The benefit of this recurrence model is the potential to identify human-derived microbes or microbial communities with prophylactic or curative properties. Bacterial communities that have coevolved within their host are likely to out-compete those from an extraneous source within that niche [12]. The use of an ^HMb^mouse model colonized with human-derived microorganisms, un-adapted to the murine environment, overcomes this inherent problem when adding back potential therapeutic human microbes [11].

## Results and discussion

### Humanized microbiota mice

**Fig. 1:**
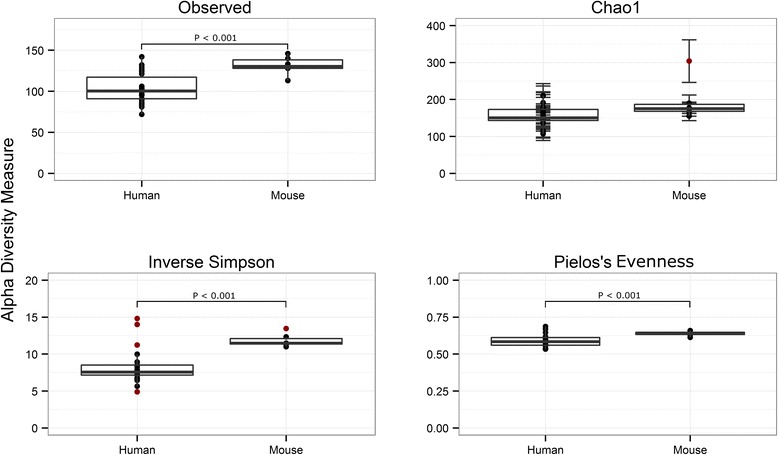
Comparison of α diversity between conventional and ^HMb^mice reveals higher diversity within conventional microbiota. A Welch two-sample *t* test was used to determine significance between the groups: observed OTUs, *p* < 0.001; Chao1 estimator (richness), *p* = 0.143; inverse Simpson (biodiversity), *p* < 0.001; and Pielou’s evenness index, *p* <0.001 (evenness of relative abundance). Holm correction applied to *p* values to account for multiple inference. Singletons were not removed for α analysis as they are required for the Chao1 estimator

To produce humanized microbiota mice (^HMb^mice), pooled human-derived feces was administered, via gavage, to germ free (GF) C57BL/6 mice. Microbiota was subsequently passed to progeny via maternal transfer and coprophagy. To monitor and compare the fecal microbial communities, we pyrosequence bar-coded and amplified bacterial V3–V5 variable region 16S rDNA in fecal samples from conventional microbiota mice (^CMb^mice, *n* = 6), HMb founder mice (GF gavaged with human feces, *n* = 6), and HMb progeny (*n* = 16). Following clustering of sequences (97 % similarity) and removal of singletons (operational taxonomic units (OTUs) that only appear once), 371 OTUs were identified across all samples (mean 3886 reads per sample). Fifty-four OTUs were shared between CMb/HMb mice with 137 unique to ^CMb^mice and 180 to ^HMb^mice. The reduced number of unique sequences found in ^CMb^mice may be attributed to the smaller sample population (*n* = 6 vs *n* = 22), limiting opportunity for species of low abundance to be sampled. This is borne out by comparison of alpha indices which show higher observed OTUs in ^CMb^mouse communities compared to ^HMb^mouse communities (mean 131.3 ± 11.4 vs 103.3 ± 18.4, *p* = <0.001). This could be anticipated as certain species from the human microbiota may not survive the transfer process or may be ill suited to the murine gastrointestinal tract. The reduction, albeit slight, is concordant with data of xenomicrobiota transferred to the murine host [13]. There was no significant difference in the Chao1 estimator, a predictor of true richness, suggesting that deeper sequencing would reveal more comparable OTU numbers. ^CMb^mouse communities possessed greater diversity (inverse Simpson index) and evenness (Pielou) of species (Fig. 1).

**Fig. 2:**
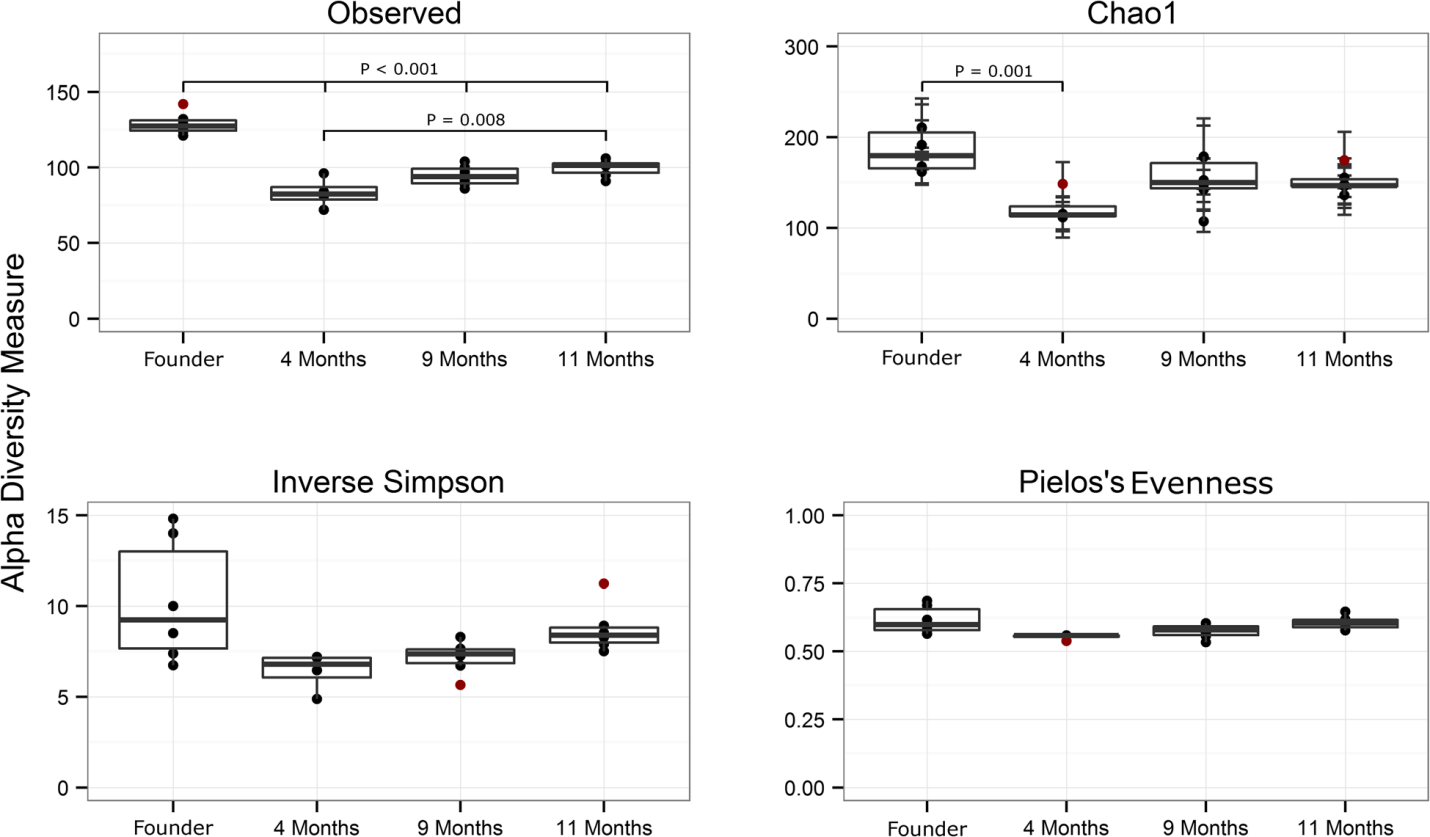
^HMb^Mice progeny have a lower α diversity than gavaged founder mice. Pairwise *t* tests with pooled SD and Holm correction for multiple tests were used to determine significant differences. Singletons were not removed for α analysis as they are required for correct calculation of the Chao1 estimator. Descendant ^HMb^mouse samples were taken from mice aged 6–10 weeks from offspring of founder mice at 4, 9, and 11 months post initial gavage of fecal material

Temporal examination of the ^HMb^mouse microbiota revealed a significant reduction in richness (though not biodiversity or evenness of relative abundance) in progeny mice compared to founder mice (gavaged fecal material). Twenty-two OTUs present in at least four of six founder mice were absent in all progeny mice (Table 1). This may indicate the inability of certain species to be transferred via the parent—offspring route or an inability of these species to remain competitive within the murine host. Following an initial drop between founder and progeny, alpha diversity trended upward over generations, though not significantly (Fig. 2).

**Table 1:** Twenty-two OTUs were present in at least four of six founder mice that were undetected in progeny, potentially indicating the inability of certain species to be transferred via the parent-offspring route

OTU	Ave. abundance^a^	% Abundance	Order	Family	Genus
0010	326	10.11	*Clostridiales*	*Ruminococcaceae*	Unclassified
0027	115.5	3.57	*Bacteroidales*	*Prevotellaceae*	*Paraprevotella*
0047	82.3	2.55	*Selenomonadales*	*Veillonellaceae*	*Megamonas*
0050	38.3	1.19	*Clostridiales*	*Lachnospiraceae*	*Lachnospiracea incertae sedis*
0061	23.5	0.73	*Bacteroidales*	*Bacteroidaceae*	*Bacteroides*
0070	17	0.53	*Clostridiales*	*Lachnospiraceae*	Unclassified
0071	16.8	0.52	*Clostridiales*	*Lachnospiraceae*	Unclassified
0072	16.8	0.52	*Clostridiales*	*Lachnospiraceae*	Unclassified
0081	14.2	0.44	*Bacteroidales*	*Rikenellaceae*	Unclassified
0087	13.2	0.41	*Clostridiales*	*Ruminococcaceae*	Unclassified
0101	9.5	0.29	*Bacteroidales*	*Porphyromonadaceae*	*Barnesiella*
0103	8.3	0.26	*Clostridiales*	*Ruminococcaceae*	*Ruminococcus*
0110	7.2	0.22	*Bacteroidales*	Unclassifed	Unclassified
0118	6.3	0.2	*Bacteroidales*	*Porphyromonadaceae*	*Barnesiella*
0124	5	0.16	*Clostridiales*	*Lachnospiraceae*	Unclassified
0128	4.5	0.14	*Clostridiales*	*Lachnospiraceae*	Unclassified
0136	3.8	0.12	*Erysipelotrichales*	*Erysipelotrichaceae*	*Erysipelotricha*
0137	3.7	0.11	*Clostridiales*	*Ruminococcaceae*	Unclassified
0163	2.7	0.08	*Fusobacteriales*	*Fusobacteriaceae*	*Fusobacterium*
0168	2.5	0.08	*Clostridiales*	*Lachnospiraceae*	Unclassified
0194	1.7	0.05	*Bacteroidales*	*Porphyromonadaceae*	*Barnesiella*
0235	1	0.03	*Erysipelotrichales*	*Erysipelotrichaceae*	*Erysipelotrichace incertae sedis*

^a^Average abundance of OTU in founder mice

To test for invasion of the ^HMb^mice communities following multiple generations under specific pathogen-free (SPF) conditions, 16S rDNA in fecal samples from ^HMb^mice (2.5 years following initial colonization of founder mice), were sequenced on the Illumina MiSeq platform and compared to the original pooled human fecal sample via reciprocal BLAST search [14]. ^HMb^Mouse communities remained remarkably immutable with ~95 % of reads from the deep sequenced ^HMb^mice matching sequences identified in the original fecal sample. Reads that could not be matched clustered into OTUs predominantly identified as *Lachnospiraceae* and may represent invasive species or those found below the level of detection in the original fecal material.

**Fig. 3:**
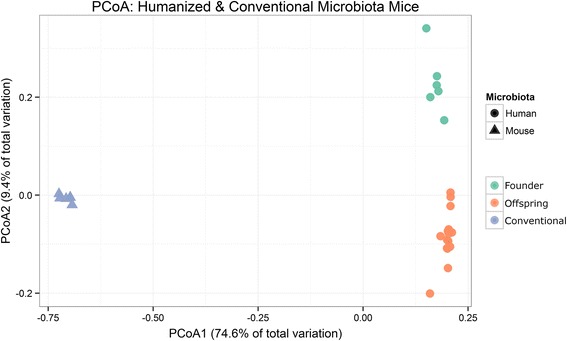
Clustering of conventional and humanized mouse microbiota based upon principal coordinates analysis (PCoA) of Bray-Curtis dissimilarities. A clear distinction can be visualized along axis 1, accounting for 74.6 % dissimilarity between conventional mouse microbiota (*blue*, *n* = 6) and humanized mouse microbiota (*green*, founder mice 3 weeks post gavage *n* = 6. *Orange*, offspring of gavaged mice with microbiota derived by maternal transfer, *n* = 16). A goodness of clustering measure, the “gap” statistic independently identified three clusters belonging to ^CMb^mice, gavaged founder ^HMb^mice, and their offspring

Visualization of the beta diversity of both CMb and HMb samples (sub-sampled to an even depth of 3224 reads per sample), using principal coordinate analysis (PCoA) of Bray-Curtis dissimilarities followed by a boot strapped Gap-Statistic analysis, identified three clusters: ^CMb^mice, gavaged founder ^HMb^mice, and progeny ^HMb^mice (Fig. 3). An independent PCoA visualization of the ^HMb^mice groups, following removal of ^CMb^mice, maintains the separation of gavaged founder ^HMb^mice. No distinction was evidenced between the multi-generation progeny (Additional file 1: Figure S1).

**Fig. 4:**
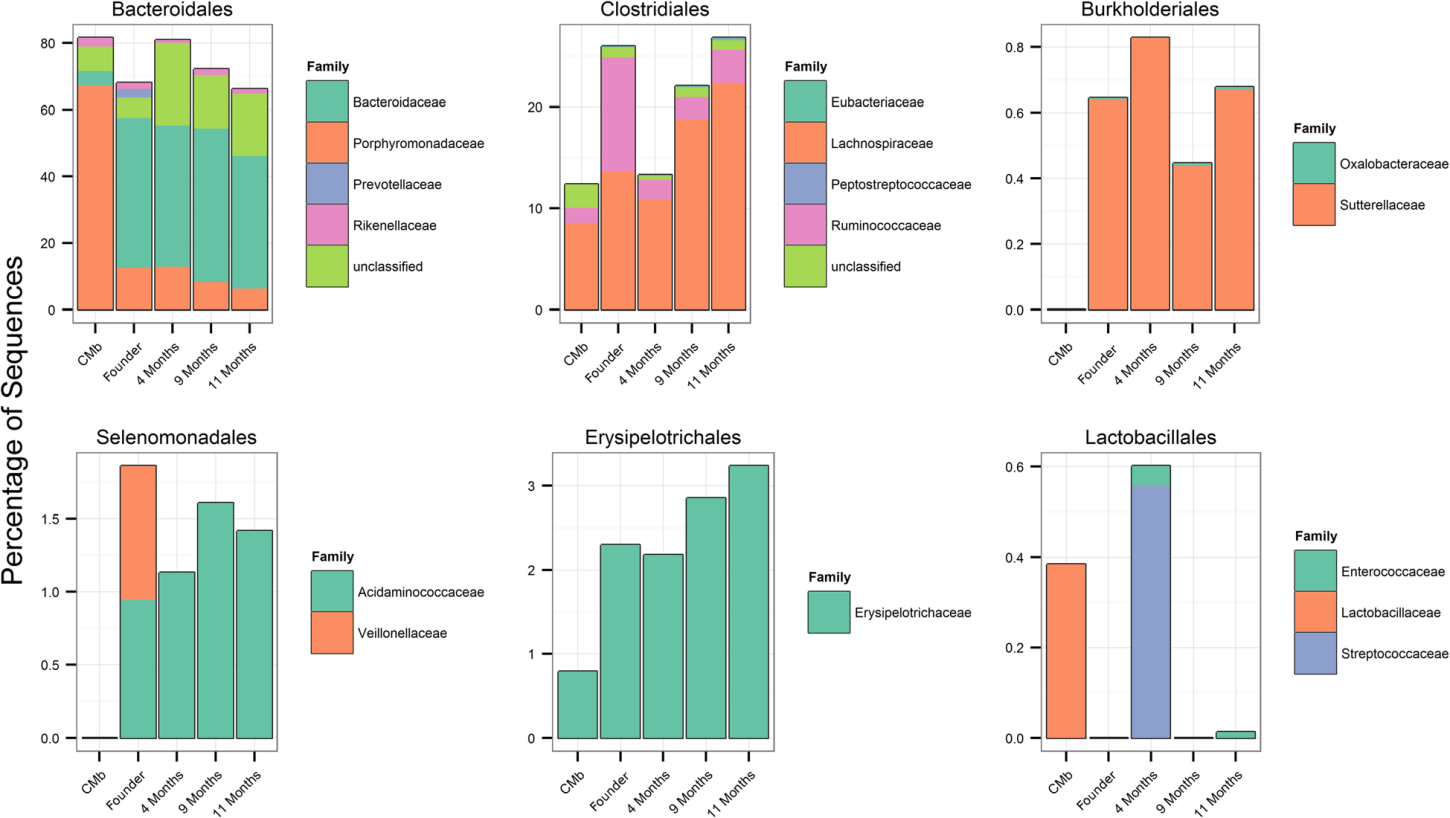
Relative abundance of major bacterial orders in the gut microbiota from CMb and HMb mice. Data are average relative abundances of OTUs assigned at the family level of sequenced communities from ^CMb^mice, *n* = 6, founder ^HMb^mice, *n* = 6; ^HMb^offspring at 4 months, *n* = 4; ^HMb^offspring at 9 months, *n* = 6; ^HMb^offspring at 11 months, *n* = 6

Similarities and differences between ^CMb/HMb^mouse communities at the family level are easily visualized in relative abundance plots (Fig. 4). The largest difference is within the *Bacteroidales*, which although similar in abundance differs markedly in its constituents between ^CMb/HMb^mouse communities. ^CMb^mouse communities are dominated by the *Porphyromondaceae* which account for almost 70 % of all sequences compared to ~10 % in ^HMb^mouse communities. Within ^HMb^mice the *Bacteroidaceae* account for the dominant group, comprising ~50 % of all sequences.

In summary, ^HMb^mice are distinguishable from ^CMb^mice by sequencing, are resistant to widespread invasion by murine species, and remain stable over multiple generations when housed under SPF conditions.

### ^HMb^Mouse *C. difficile* infection model

**Fig. 5:**
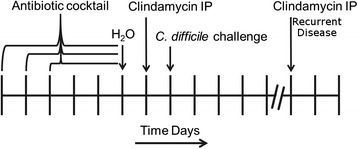
Experiment design. A five-antibiotic cocktail consisting of kanamycin (0.4 mg ml^−1^), gentamicin (0.035 mg ml^−1^), colistin (850 U ml^−1^), metronidazole (0.215 mg ml^−1^), and vancomycin (0.045 mg ml^−1^)) was administered ad libitum in drinking water for 3, 4, or 5 days. Antibiotic water was changed to sterile antibiotic free water and 24 h later mice were administered an intra-peritoneal injection of clindamycin (10 mg kg^−1^). Twenty-four hours post clindamycin IP, mice are challenged with *C. difficile* spores by oral gavage and monitored for signs of disease. Recurrent CDI is induced by administration of a further clindamycin injection (10 mg kg^−1^)

**Fig. 6:**
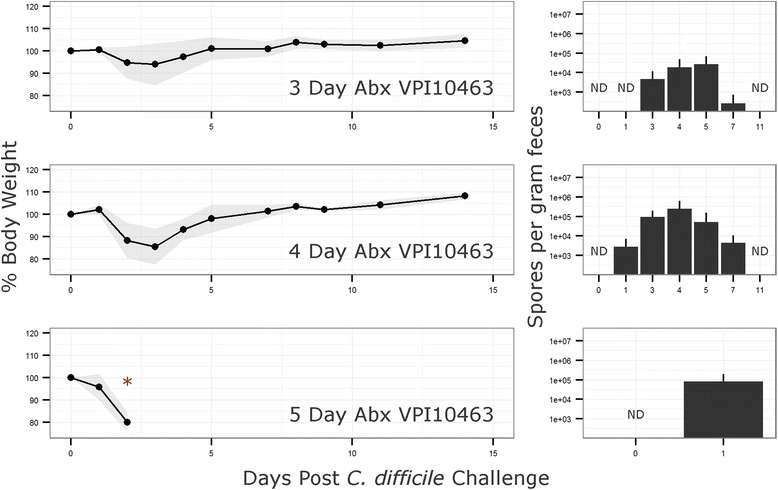
Mean relative mouse weights and spore counts after *C. difficile* VPI10463 challenge. Mice were administered a five-antibiotic cocktail in drinking water ad libitum for 3, 4, or 5 days followed by an IP injection of clindamycin (10 mg kg^−1^). Twenty-four hours after IP injection, mice were challenged with the high toxin producing strain VPI10463 (*n* = 6 for each group). Antibiotic and *C. difficile* control groups showed no signs of weight loss or disease (data not shown). Fecal samples were homogenized in 500 μl PBS and heated to 65 °C for 30 min to kill vegetative cells. Samples were serially diluted and plated in replicate on TCCFA plates to enumerate *C. difficile* spore numbers. No spores were detected in the negative control group or the no antibiotics control group even with 10^6^ spores administered by gavage (not shown). *Shaded area* and *error bars* indicate standard deviation from mean. *6/6 mice became moribund. *ND* spores non-detectable

**Fig. 7:**
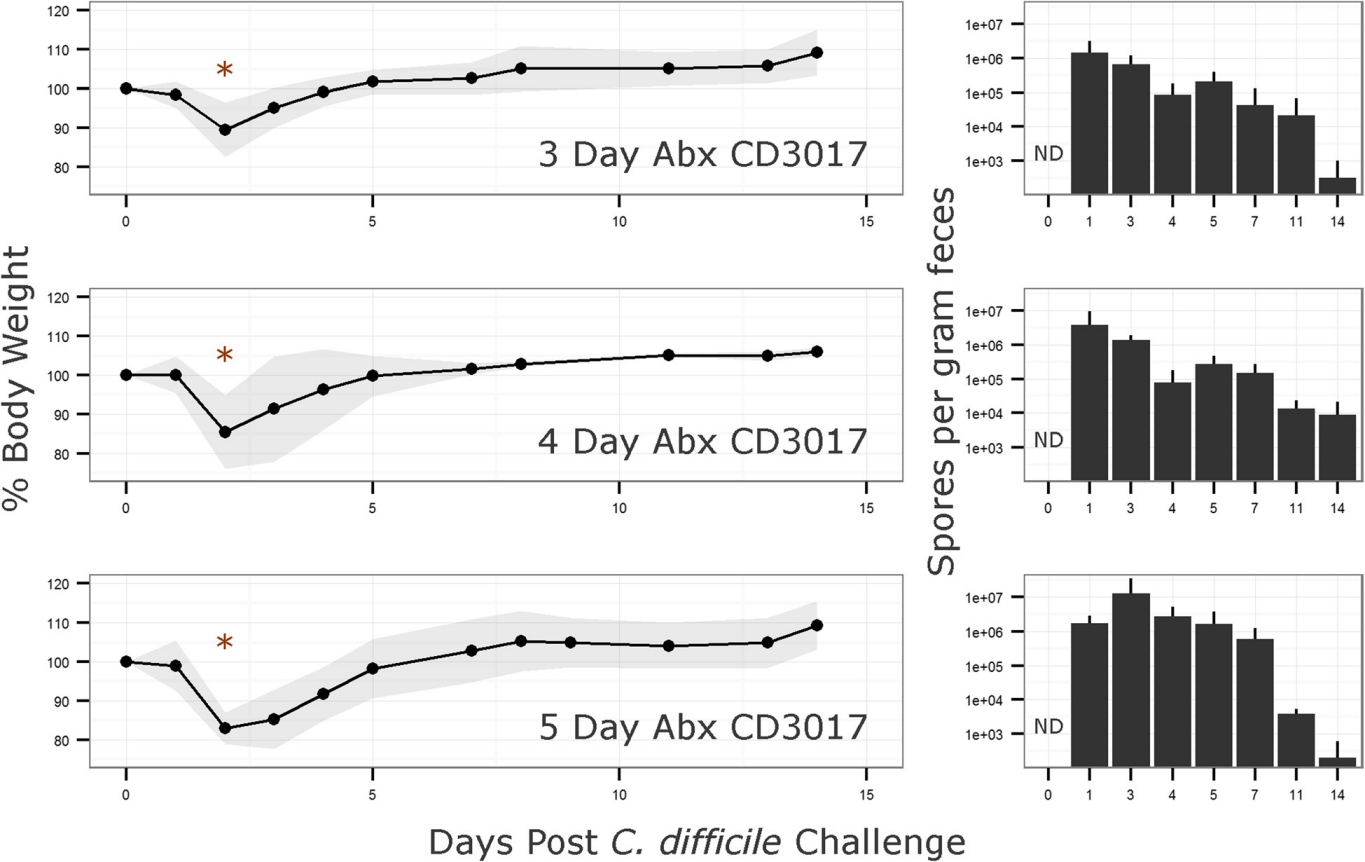
Mean relative mouse weights and spore counts after *C. difficile* CD3017 challenge. Mice were administered a five-antibiotic cocktail in drinking water ad libitum for 3, 4, or 5 days followed by an IP injection of clindamycin (10 mg kg^−1^). Twenty-four hours after IP injection mice were challenged with the clinical NAP1/B1/027 ribotype strain, CD3017 (*n* = 6 for each group). Fecal samples were homogenized in 500 μl PBS and heated to 65 °C for 30 min to kill vegetative cells. Samples were serially diluted and plated in replicate on TCCFA plates to enumerate *C. difficile* spore numbers. *Shaded area* and *error bars* indicate standard deviation from mean. *1/6, 3/6, and 2/6 mice became moribund in the 3-, 4-, and 5-day Abx groups, respectively. *ND* spores non-detectable

Models of *C. difficile* infection, initially developed in the Syrian hamster [15], have been established in conventional and gnotobiotic mice as well as other animals encompassing a broad host range [6, 16]. To produce a robust infection model in stably colonized ^HMb^mice, a number of antibiotic pretreatment regimes were tested (see “Methods” section). Only the use of a five-antibiotic cocktail administered ad libitum in drinking water followed by intraperitoneal (IP) injection of clindamycin resulted in consistent results. To examine whether the duration of antibiotic pretreatment affected *C. difficile* disease susceptibility or severity, ^HMb^mice were administered the five-antibiotic cocktail for a period of 3, 4, or 5 days followed by clindamycin IP injection. Mice were challenged 24 h later with 5 × 10^5^ spores of either the high toxin producing lab strain VPI10463 or a clinically relevant NAP1/B1/027 strain CD3017 [8] (see experimental outline, Fig. 5). Disease severity, measured here by maximum weight loss (48 h post challenge), was significantly different across antibiotic pretreatment groups when challenged with VPI10463 (Kruskal-Wallis test, *χ*^2^ = 9:84, *p* = 0.007; post hoc Mann-Whitney tests with Holm correction: 3 vs 5 days, *p* = 0.015; Fig. 6). Furthermore, all animals in the 5-day antibiotic pretreatment (*n* = 6) reached a moribund state requiring euthanasia, whereas animals in the 3- (*n* = 6) and 4 (*n* = 6)-day groups recovered. Of the mice challenged with CD3017, 6 of 18 (1/6, 3/6, and 2/6 from the 3-, 4-, and 5-dayantibiotic treatment groups, respectively) reached a moribund state within 48 h. Weight loss was higher in the groups with longer antibiotic treatment; however, due to the reduced number of mice (and therefore statistical power), the difference was not significant (Fig. 7).

A Kruskal-Wallis test on spore count data identified a significant difference in VPI10463 spore numbers 24 h post challenge between antibiotic pretreatment groups (*χ*^2^ = 12:91, *p* = 0.002). Post hoc Mann-Whitney tests with Holm correction established significant differences between groups 3 day (below level of detection) and 5 day (mean = 8.00 × 10^4^ spores g^−1^ feces), *p* = 0.008, and groups 4 day (mean = 2.78 × 10^3^ spores g^−1^ feces) and 5 day, *p* = 0.023. After 48 h, only mice in the 3- and 4-day antibiotic pretreatment groups survived with no significant difference in spore counts (Fig. 6). Mice challenged with CD3017 did not have a significant difference in spore production between antibiotic pretreatment groups (Fig. 7). However, spore levels were higher in mice that became moribund compared to those that recovered (mean = 4.61 × 10^6^ vs mean = 1.41 × 10^6^ spores g^−1^ feces), though this difference was not statistically significant due to a large standard deviation from the mean. Across all time points sampled, CD3017 consistently produced more spores than VPI10463 and continued producing detectable spore levels for a longer period of time. CD3017 spores were observable 14 days post challenge (mean across all CD3017 groups day 14 = 4.42 × 10^3^ spores g^−1^ feces), whereas VPI10463 spores were undetectable by day 11. The VPI10463 strain is known to be a poor spore former both in vitro [17] and in vivo [18]. Conversely, increased spore production has been proposed as a mechanism for the spread of NAP1/B1/027 strains [19], though this is controversial with a wide range of sporulation efficiencies seen in vitro [20].

**Fig. 8:**
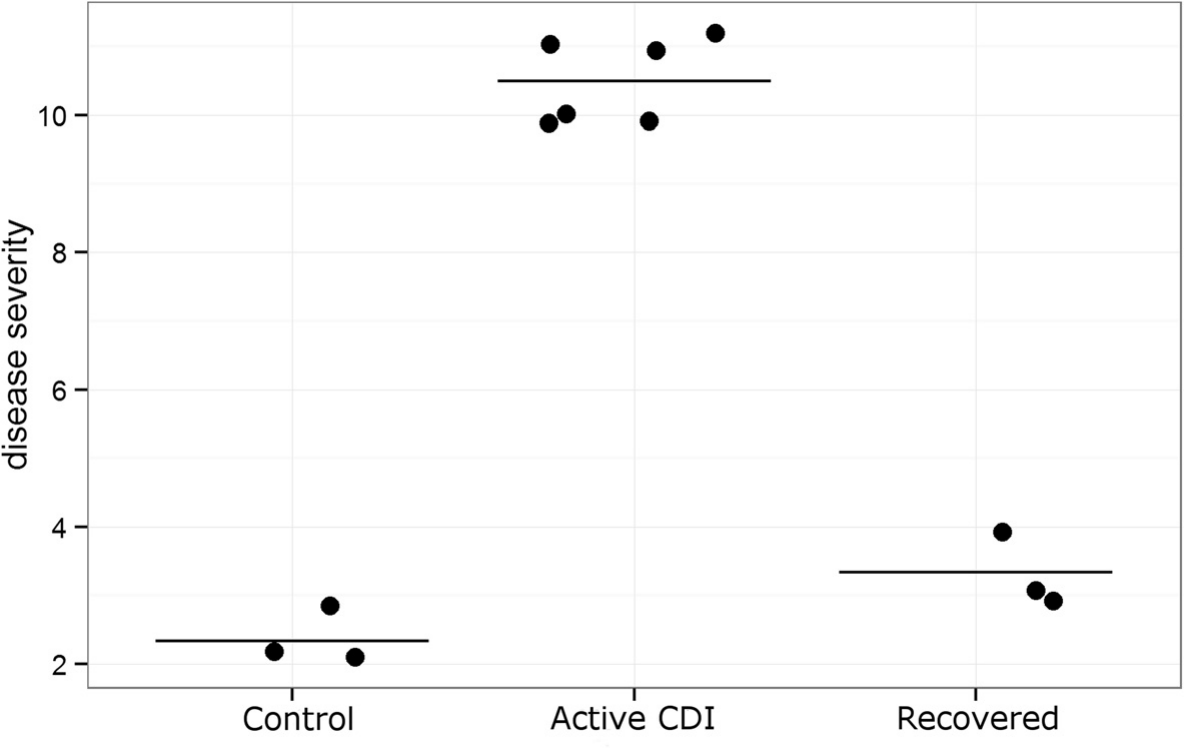
Histopathology of disease. Histological sections of distal colon were scored blindly by an independent researcher. Three criteria, edema, cellular infiltration, and epithelial damage, were individually scored from 0–4 (a score of 0 indicates no pathology), and the three values summed to give a disease severity score

To examine the effect of tissue damage within the ^HMb^mice, histopathology was undertaken on portions of the distal colon. Samples were scored for edema, cellular infiltration, and epithelial damage on a scale of 0–4 for each metric (0 being no abnormality). The sum of scores (maximum of 12) was taken as an indicator of overall severity. Control mice that received no antibiotic pretreatment (i.e., were not susceptible to CDI) but were administered 5 × 10^5^ VPI10463 spores or that received antibiotic treatment without *C. difficile* challenge showed minimal pathology (*n* = 3, mean score 2.33 ± 0.58); mice that had recovered from CDI presented mild disease signs (*n* = 3, mean score 3.33 ± 0.58) while moribund mice had severe intestinal pathology (*n* = 6, mean score 10.5 ± 0.55) (Fig. 8).

### Recurrent model of CDI

**Fig. 9:**
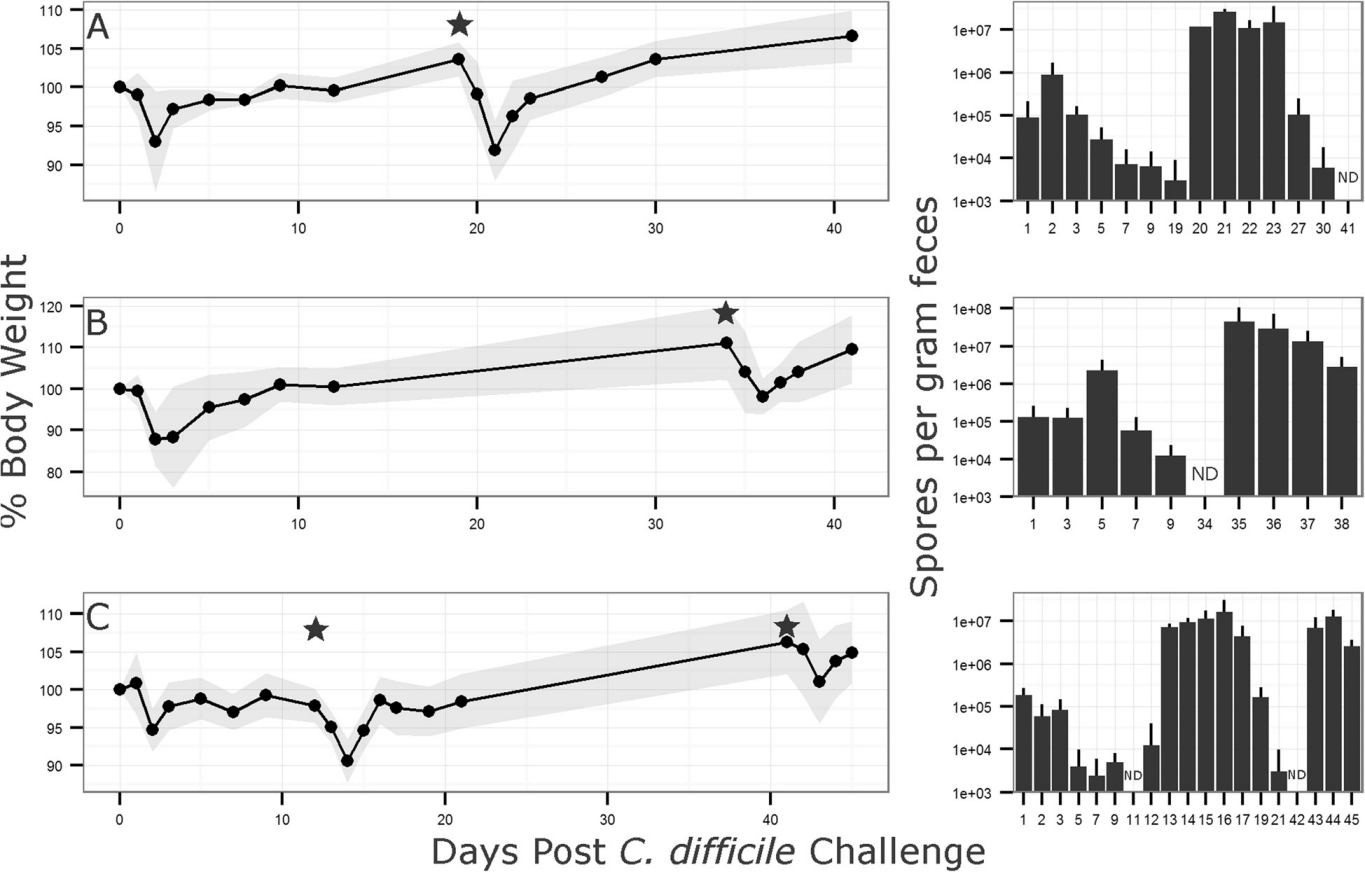
^HMb^Mouse model of recurrent CDI. Mice were administered a five-antibiotic cocktail in drinking water ad libitum for 4 days followed by an IP injection of clindamycin (10 mg kg^−1^). Twenty-four hours after IP injection, mice were challenged with the clinical NAP1/B1/027 ribotype strain, CD3017 (**a**: *n* = 5, **b**: *n* = 4, **c**: *n* = 4). Relapse was induced by further IP injection of clindamycin (10 mg kg^−1^). *Shaded area* and *error bars* indicate standard deviation from mean. *Star* = clindamycin-induced relapse. *ND* spores non-detectable. *C. difficile* Spore numbers were enumerated by homogenizing fecal samples in 500 μl PBS and heating to 65 °C for 30 min to kill vegetative cells followed by serial dilution and plating in replicate on TCCFA plates

Recurrent CDI is a considerable complication of infection, occurring in as many as 25 % of patients. In a separate experiment, ^HMb^mice were challenged with 5 × 10^4^ CD3017 spores following 4 days of the five-antibiotic pretreatment and clindamycin IP injection. The reduced number of spores administered resulted in less weight loss across all mice during the initial infection (*n* = 13, mean % bodyweight lost 48 h post challenge = 8.16 % ± 5.9). Mice were allowed to recover from signs of disease (weight loss, detectable spores). After recovery from initial infection, a recurrence of CDI was triggered by a single IP injection of clindamycin. No additional spores were administered. Groups of mice were found to be susceptible to relapse when administered clindamycin 11, 19, or 35 days after the first *C. difficile* challenge (Fig. 9a–c). ^HMb^Mouse weight immediately prior to relapse was normalized to 100 %. Weight loss did not significantly differ between groups upon relapse, regardless of when triggered (11–35 days post initial challenge, combined weight loss 48 h after relapse: *n* = 13, 9.87 % ± 3.43). Following an initial clindamycin induced relapse (11 days postinfection), a further CDI episode could be provoked (41 days post the initial infection and 30 days after the first relapse) upon an additional clindamycin injection (*n* = 5, mean weight loss 48 h post second relapse 4.97 % ± 2.11) (Fig. 9c).

*C. difficile* spores in feces were below the level of detection at the time of relapse induction (as determined by plating). One possibility is that spores adhered to the coat of ^HMb^mice (cages were changed with sterile replacements weekly), triggering relapse upon ingestion in post clindamycin-susceptible animals. Another hypothesis is that vegetative cells or spores remained associated, at low levels, within the murine gastrointestinal (GI) tract. Within 24 h of triggering CDI relapse, spores were detected at levels ≥10^7^ g^−1^ feces, where they remained for ~5 days before waning to undetectable levels (Fig. 9). This rapid increase suggests that vegetative cells may already be present within the intestine, allowing for rapid growth and spore production when conditions permit. An association of *C. difficile* and the gut mucosa/epithelium has been observed previously in both animal [21] and in vitro experiments [22].

### Effect of antibiotics and *C. difficile* infection on the ^HMb^mouse microbiota

The use of antibiotics can significantly reduce or ablate sensitive species while simultaneously allowing resistant opportunists to bloom [23]. To examine this effect in ^HMb^mice, fecal samples were sequenced by Illumina MiSeq (average reads per sample 35,505, sub-sampled to an even depth of 23,057) from three time points: base composition, prior to antibiotic insult; post-antibiotics, immediately following the five-antibiotic cocktail and clindamycin IP injection; recovered, following challenge with *C. difficile* and subsequent recovery from CDI, 14–17 days post cessation of antibiotics.

**Fig. 10:**
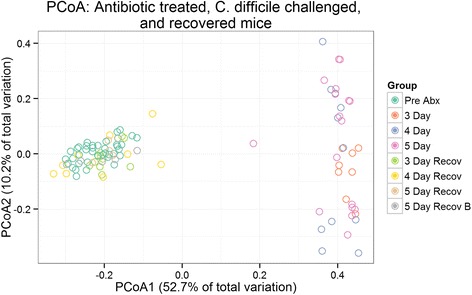
Clustering of ^HMb^mice communities: pre-antibiotics, post-antibiotics, and following recovery from *C. difficile* infection based upon principal coordinates analysis (PCoA) of Bray-Curtis dissimilarities. ^HMb^Mouse communities that have undergone 3, 4, or 5 days of antibiotic treatment (3 day, 4 day, 5 day) separate clearly from pre-antibiotic or recovered groups, though do not form distinct clusters of their own as determined by the gap statistic. Communities that have recovered from antibiotic assault only (5 day recov B) and antibiotic assault plus *C. difficile* invasion (3 day recov, 4 day recov, 5 day recov) return to a pre-antibiotic-like state as evidenced by the close grouping of these communities

Comparison of microbial community structures for changes in abundance enables identification of bacteria that potentially facilitate susceptibility/increased severity or resistance/amelioration of *C. difficile* disease. Principal coordinate analysis of Bray-Curtis dissimilarity of communities prior to antibiotics (*n* = 38), following 3, 4, or 5 days of antibiotics (*n* = 36), and ensuing recovery from *C. difficile* infection (*n* = 31) was undertaken. Examination of communities from the antibiotic time courses revealed little obvious difference in community structure regardless of duration. Furthermore, communities that recovered from the antibiotic assault (14–17 days post-antibiotic cessation) and *C. difficile* challenge look remarkably similar to unperturbed, pre-antibiotic, communities (Fig. 10) despite their susceptibility to clindamycin-induced CDI relapse.

### Pre- vs post-antibiotic treated microbiota

To examine the effect of antibiotics on the ^HMb^mouse microbiome in more detail, data were subset into pre-antibiotic and immediately post-antibiotic cessation samples. Differentially abundant OTUs between the two groups were detected using negative binomial generalized linear models with DESeq2 in R. Originally designed to detect differentially abundant gene expression from RNA-Seq data, this package has been shown to work well with 16S sequencing count data [24]. *p* values were corrected for multiple inference using the Benjamini-Hochberg false discovery rate (FDR) procedure and a strict adjusted alpha cutoff value of 0.01 set as criteria for inclusion. Two hundred and eighteen OTUs were found to have significantly different abundances. One hundred and ninety-eight OTUs decreased while 20 OTUs increased post-antibiotic use (see Additional file 2: Table S1). Of those OTUs that decreased, 44.4 % belonged to the *Lachnospiraceae*, 16.7 % to the *Ruminococcaceae*, 9.1 % *Bacteroidaceae*, and the remaining 29.8 % were split across 12 family groups or unclassified. This wide-ranging reduction of taxa can be explained by the fact that the five-antibiotic cocktail possesses a broad spectrum of antimicrobial activity, targeting both Gram positive and negative species via multiple modes of action.

Proposed mechanisms of colonization resistance in respect to *C. difficile* include competition for sparse resources within a niche; microbially produced short-chain fatty acids, specifically butyrate; and the conversion of primary bile acids into secondary acids via 7α-dehydroxylating bacteria. Overall, 69.7 % of the OTUs significantly decreased by antibiotic treatment belonged to the *Clostridia*, potentially freeing a niche for *C. difficile* to exploit. Butyrate possesses anti-inflammatory properties, provides the preferred energy source in colonocytes, and can directly inhibit *C. difficile* growth in vitro [25]. Members of the *Lachnospiraceae* and *Ruminococcaceae* are known to be primary butyrate producers [26], so their significant reduction likely leads to reduced butyrate production, increasing susceptibility to CDI. Primary bile acids, such as taurocholic acid, are known to induce *C. difficile* germination while secondary bile acids can exert inhibitory effects on germination or kill vegetative cells [27]. The majority of known bile acid 7α-dehydroxylating bacteria belong to *Clostridium* cluster XIVa [28], of which six OTUs were significantly reduced following antibiotics. Removal of 7α-dehydroxylating bacteria can alter the proportions of primary and secondary bile acids, switching the environment from inhibitory to stimulatory in respect to *C. difficile* spore germination [29, 30].

Of the OTUs that increased, the largest increase (base mean = 159, log_2_ fold change = 8.48) belonged to an *Enterococcaceae* which are known to bloom in humans undergoing certain antibiotic treatments [31, 32] and have been shown to correlate positively with CDI [33]. These blooms may be incidental or may have a synergistic effect on disease severity.

### Microbiota in moribund vs recovered mice

Indicator species analysis can be applied to identify OTUs that differ in their occurrence or relative abundances between groups. The fidelity (probability an OTU occurs within a group) and specificity (the proportion of groups that contain an OTU) of all OTUs were calculated and the statistical significance of their associations inferred by permutation followed by Benjamini-Hochberg correction for multiple inference [34]. An OTU is highly characteristic of a group if it is significantly more likely to occur in that group than in another or is considerably more abundant in that group. Mice challenged with *C. difficile* following antibiotic pretreatment can be split into two groups: “alive” (those that recover) and “dead” (those that become moribund). By carrying out indicator species analysis on these groups, it is possible to correlate OTUs to outcomes that, with further empirical testing, may be shown to reduce or increase the effect of CDI. Four OTUs were associated with mice that survived and 13 with moribund animals (Table 2, alpha cutoff for inclusion = 0.1). Of the four OTUs indicative of mice that survived, two stood out: OTU44, classified to the genus level as *Clostridium* cluster XIVa, a group associated with butyrate production [35]; and OTU91, a *Blautia* whose sequence matched *Blautia hansenii* by BLAST search and is annotated to possess bile-acid 7α-dehydroxylase activity. Recently, *Blautia hansenii* was predicted to have a negative, inhibitory, interaction with *C. difficile* in both humans and mouse models [36]. Furthermore, the addition of a bacterial species, *Clostridium scindens*, containing bile-acid 7α-dehydroxylase activity was shown to ameliorate CDI in a mouse model [36]. No obvious associations could be made for OTUs indicative to moribund animals, in part because eight of the 13 were unclassified at the family level. Those that were identified comprised *Parabacteroides* (2), *Parasutterella*, *Clostridium sensu stricto*, and an unclassified *Ruminococcaceae*. The isolation and testing of these strains for their protective, in the first instance, or synergistic deleterious effect, in the latter, may provide further insight into the treatment or progression of CDI.

**Table 2:** Differentially abundant OTUs in moribund vs recovered ^HMb^mice communities

OTU	Association	padj	Order	Family	Genus
00091	Alive	0.065	*Clostridiales*	*Lachnospiraceae*	*Blautia*
00069	Alive	0.0041	*Clostridiales*	*Lachnospiraceae*	Unclassified
00044	Alive	0.0313	*Clostridiales*	*Lachnospiraceae*	*Clostridium* XIVa
00106	Alive	0.0611	*Bacteroidales*	Unclassified	Unclassified
00172	Dead	0.0351	Unclassified	Unclassified	Unclassified
00377	Dead	0.0134	*Clostridiales*	Unclassified	Unclassified
00127	Dead	0.0279	Unclassified	Unclassified	Unclassified
00257	Dead	0.0282	Unclassified	Unclassified	Unclassified
00153	Dead	0.0406	*Clostridiales*	Unclassified	Unclassified
00201	Dead	0.0392	*Bacteroidales*	*Porphyromonadaceae*	*Parabacteroides*
00189	Dead	0.0286	*Bacteroidales*	*Porphyromonadaceae*	*Parabacteroides*
00250	Dead	0.0566	*Bacteroidales*	Unclassified	Unclassified
00441	Dead	0.061	*Burkholderiales*	*Sutterellaceae*	*Parasutterella*
00769	Dead	0.0558	*Clostridiales*	*Clostridiaceae*	*Clostridium sensu stricto*
00195	Dead	0.0407	*Clostridiales*	Unclassified	Unclassified
00064	Dead	0.0755	*Clostridiales*	*Ruminococcaceae*	Unclassified
01786	Dead	0.0536	Unclassified	Unclassified	Unclassified

Mice challenged with 5 × 10^6^ CD3017 spores following antibiotic pretreatment were parsed into two groups: moribund (those mice that required euthanasia, *n* = 4) and recovered (mice that survived CDI, *n* = 9) with the differential abundances of OTUs immediately prior to *C. difficile* infection analyzed using DESeq2 in R. *p* values were corrected using the Benjamini-Hochberg false discovery rate (FDR) procedure and a corrected alpha value cutoff of <0.1 used for inclusion

### Recovery of ^HMb^mouse microbiota following antibiotic treatment and *C. difficile* infection

**Fig. 11:**
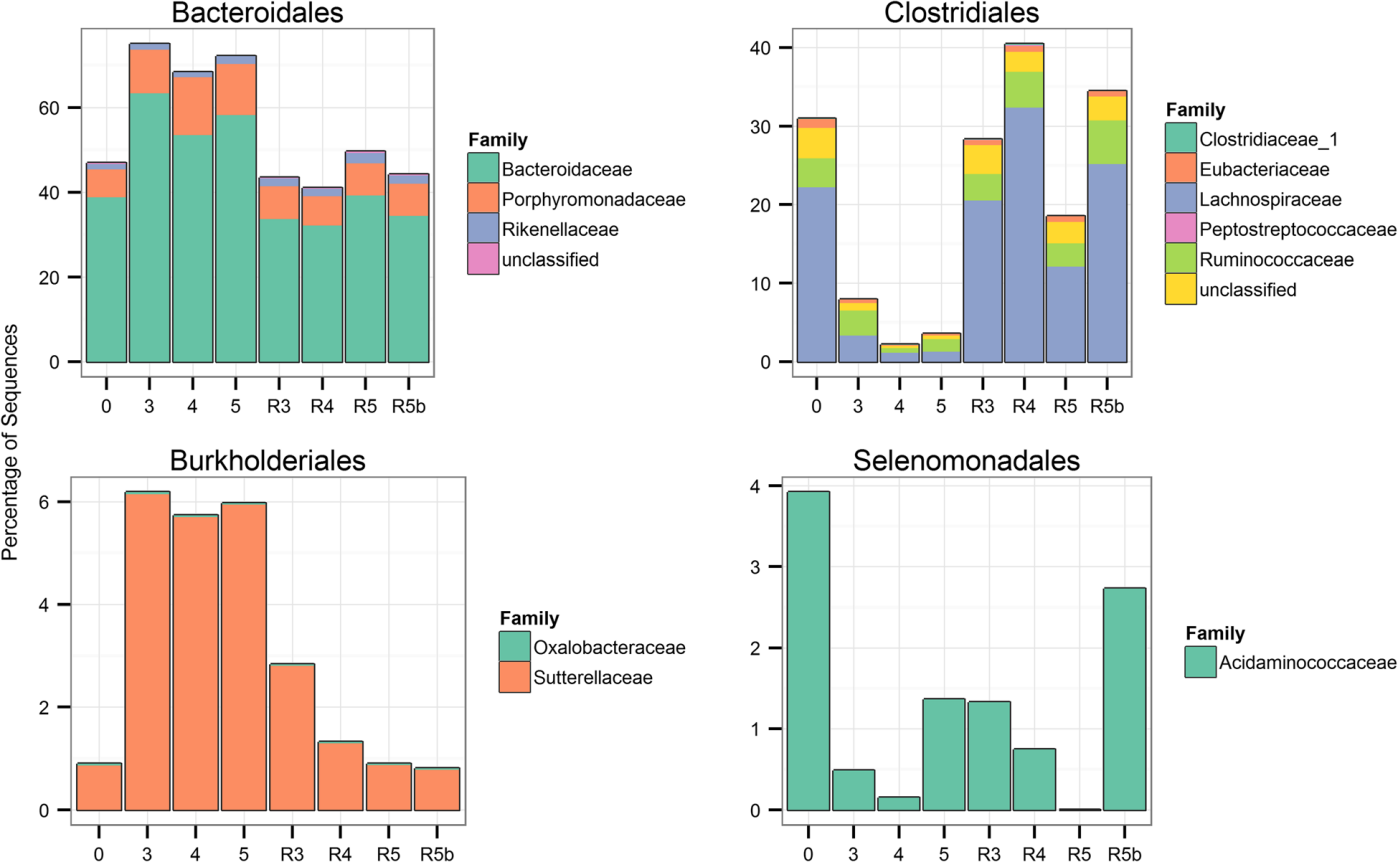
Relative abundance of major bacterial orders broken down at family level in the gut microbiota from ^HMb^mice. Data are average relative abundances of OTUs assigned at the family level of sequenced communities from: 0, pre-antibiotics (*n* = 38); 3, 3 day Abx (*n* = 8); 4, 4 day Abx (*n* = 11); 5, 5 day Abx (*n* = 17), R3, 3 day Abx group 14–17 days postinfection (*n* = 12); R4, 4 day Abx group 14–17 days postinfection (*n* = 9); R5, 4 day Abx group 14–17 days postinfection (*n* = 4); R5b, 5 day Abx group 14–17 days post cessation of antibiotics—no *C. difficile* challenge (*n* = 6)

It has been proposed that just a single course of antibiotic use can lead to long-term shifts in the microbiota [37]. Visualization of family level abundances reveals similarity between the untreated and the recovered groups suggesting that ^HMb^mice communities return to their stable state within 14–17 days of antibiotic cessation (Fig. 11). To examine the effect of antibiotic treatment followed by CDI in ^HMb^mice communities, the data were subset into pre-antibiotic (*n* = 38) and recovered (*n* = 25) samples (taken 14–17 days post-antibiotic cessation and *C. difficile* challenge); samples that received antibiotics but were not challenged with *C. difficile* were excluded from analysis. Forty-two OTUs showed a significant difference, considerably less than the 218 in the pre vs post antibiotic treatment groups, including a decrease in 19 OTUs and an increase in 23 OTUs in the recovered communities compared to the pre-antibiotic-treated communities (Additional file 3: Table S2). Eighteen of the 19 OTUs with reduced abundance were also diminished immediately following antibiotic cessation indicating that they had not yet recovered, though in all cases were trending back towards their base abundance (i.e., the log_2_ fold change was closer to zero). Just one OTU, classifying as a *Clostridium* cluster XlVb, had reduced abundance in the recovered samples that was not seen to be reduced immediately following antibiotic cessation. Of the 23 OTUs with increased abundance, 14 were OTUs that have been significantly reduced by antibiotics and had rebounded during recovery to a higher than baseline state. Of the remaining OTUs, one, present in 18 of 25 recovered samples, was *C. difficile* as determined by BLAST search. The presence of *C. difficile*, albeit at low levels (0–24 reads in non-sub-sampled/normalized samples or 0–0.07 % of reads, median 0.003 %), is evidence that it is not always cleared following recovery from disease, providing a potential reservoir for relapse when conditions permit. The remaining eight OTUs were diversely composed of *Lachnospiraceae*, *Bacteroidaceae*, *Streptococcaceae*, *Clostridiaceae*, and *Verrucomicrobiaceae*. Examination of the alpha metrics associated with pre-antibiotic and recovered samples show a significant increase in estimated richness while evenness is reduced in recovered samples, though the difference in both cases is slight. There was no disparity in diversity between the two groups (Table 3). Taken together, it could be concluded that ^HMb^mice communities would return to their base constitution given enough time following antibiotic insult.

**Table 3:** Comparison of alpha measurements between pre-antibiotic and recovered ^HMb^mouse communities

		Observed			Chao1			Inverse Simpson			Pielou’s evenness		
Group	*N*	Mean	SD	*P*	Mean	SD	*P*	Mean	SD	*P*	Mean	SD	*P*
Pre Abx	38	191.9	26	0.051	265.7	57.7	0.006	12.6	3.3	0.631	0.612	0.03	0.009
Recov	25	205.7	27.2		315.3	71.6		12.2	2.8		0.59	0.03	

*SD* standard deviation. *P* student’s *t* test with Holm adjustment for multiple comparisons

## Conclusion

Fecal microbial transplantation (FMT) as a treatment for *C. difficile* disease is not a contemporary idea, having been reported in scientific literature for over 50 years [38]. Recently, the use of FMT for the treatment of recurrent CDI has burgeoned with a reported efficacy of >90 % [4]. Despite this, CDI remains the most prevalent nosocomal disease in the developed world. There are tangible risks associated with administering an unknown microbiota to a patient. Increased use of FMT will bring with it a heightened likelihood of seeing complications such as infection, allergic response, or long-term unsuspected sequelae such as weight gain [39]. An alternative to using the mélange of bacteria, fungi, and virons present in FMT samples is to create a defined cocktail of well characterized, sequenced, bacteria that is shown to be efficacious in its treatment or prevention of CDI. This approach has been successfully applied in conventional mouse models using indigenous murine microbes [11] and to ameliorate disease using specific human derived microbiota [36].

The use of ^HMb^mice as a model of both CDI and recurrent CDI in combination with sequencing technology and advanced bacterial isolation techniques provides a resource for the identification of beneficial microbes and the means to test them among other human-derived microbes.

## Methods

### Humanized microbiota mice

Germ-free C57BL/6 mice were administered, via oral gavage, a human-derived fecal microbiota from a pooled fecal slurry of 12 healthy adults as described in Robinson et al. [8] and classified as ^HMb^mice. ^HMb^mice derived by gavage were housed and bred in gnotobiotic isolators. Progeny and descendants of the founder ^HMb^mice acquired their microbiota via maternal transfer and were maintained under SPF conditions. To limit the introduction of invasive species, gloves were changed between handling conventional microbiota mice (^CMb^mice) and all chow, water, and bedding autoclaved prior to use. All descendant ^HMb^mouse samples were taken from mice aged 6–10 weeks from offspring of founder mice at time points 4, 9, and 11 months post initial gavage of fecal material. A separate sample was taken from mice 2.5 years, and multiple generations, out for comparison to the original starting fecal material. The animal use protocol was approved by the Animal Ethics Committee of Baylor College of Medicine (Protocol no. AN-6675).

### Microbiome analysis

Mouse fecal samples were collected in sterile microfuge tubes and stored at −80 °C until needed. DNA was extracted by bead beating and modified extraction and cleanup with the Qiagen DNEasy Tissue Kit as described previously [8]. For temporal analysis of the ^HMb^mice, the V3–V5 region of the 16S rRNA gene was amplified by PCR using the 357F/962R primers with unique barcodes designed by the Human Microbiome Project [40] and sequenced using a 454 GS Junior (Roche Diagnostics). Analysis of microbial communities following antibiotic administration and *C. difficile* challenge experiments was carried out by amplifying the V4 region of the 16S rRNA gene with primers F515/R806, using a dual indexing approach (4 forward primer; 96 reverse primer) and subsequent sequencing by Illumina MiSeq. The 96-indexed R806 primers used were previously described in Caparaso et al. [41] (806rbc0–806rbc96). The indexed F515 primers were essentially as described in Kozich et al. [42], except that we generated four barcodes that balanced the nucleotide composition at each position (atcgatgg, tcacgaca, ggtatctc, and cagtcgat) in place of those described in Kozich et al.

PCR reactions were carried out in triplicate. Each reaction contained 4 μl of diluted template, 1X Phusion High-Fidelity Buffer (New England Biolabs), 200 μM dNTPs (Promega or Invitrogen), 10 nM primers, 0.2 units of Phusion DNA Polymerase (New England Biolabs), and PCR grade water to a final volume of 20 μl. The amplification cycle consisted of an initial denaturation at 98 °C for 30 s, followed by 30 cycles of 10 s at 98 °C, 20 s at 51 °C, and 1 min at 72 °C. Replicates were pooled and cleaned using AMPure beads as previously described [8]. DNA sample concentrations were determined with Quant-iT (Life Technologies) and pooled at equimolar ratios. Prior to sequencing, the quality of pooled DNA was assessed by analysis on a Bioanalyzer High Sensitivity DNA Kit (Agilent).

### Sequence data analysis

Sequence data were processed using platform-specific (454/MiSeq) pipelines in Mothur v.1.34.3 [43] based upon published SOPs [44, 45]. Alignment was achieved using the Silva 16S rRNA gene reference database. Chimeric sequences and any classifying as chloroplast, mitochondria, Archaea, or Eukaryota were removed after identification by the Mothur implementation of uchime or mothur-formatted ribosomal database project (version 9) classifier, respectively. Sequences were clustered by the average-neighbor method into operational taxonomic units (OTUs) of ≥97 % sequence identity. Analysis of OTU data were performed in R [46] using the phyloseq [47], DESeq2 [48], and indicspecies [49] packages. Sequence data are available via the Sequence Read Archive (SRA) accession numbers SRP061089 and SRP061088.

### Alpha diversity

To examine differences in community alpha measurements, several metrics were compared. Richness: both a count of observed OTUs and an estimate of true species richness of a sample with Chao1. Evenness: a measure of how similarly taxa are represented in abundance. Pielou’s evenness index ranges from zero to one with low values indicating uneven communities in which one or a few taxa are highly represented and high values indicating communities with more even representation among taxa. Diversity: the quantification of both richness and evenness. The inverse Simpson index values are higher when either many taxa are present, or when they are more similar in abundance.

$$ \mathrm{Chao}1\ :\ {S}_1={S}_{\mathrm{obs}}+\frac{F_1^2}{2{F}_2} $$

$$ \mathrm{Pielou}\hbox{'}\mathrm{s}\kern0.5em \mathrm{evenness}\kern0.5em :\kern0.5em J = \frac{-{\Sigma}_{i-1}^{S_{\mathrm{obs}}} pi \ln\ pi}{ \ln \left({S}_{\mathrm{obs}}\right)} $$

$$ \mathrm{Inverse}\ \mathrm{Simpson}\ :\ \frac{1}{D}\ \mathrm{where}\ D = \frac{\Sigma_{i-1}^{S_{\mathrm{obs}}}ni\left(ni-1\right)}{N\left(N-1\right)} $$

*S*_obs_ = the number of species in the sample, *F*_1_ = the number of singletons (single occurrence within a sample) and *F*_2_ = the number of doubletons.

*pi* = proportion of S_obs_ made up of the *i*th species.

*ni* = the number of individuals in the *i*th OTU and *N* = the total number of individuals in the community.

### Beta diversity

The Bray-Curtis dissimilarity matrix was used to describe differences in microbial community structure. Patterns of similarity among the different ^HMb/CMb^mouse fecal communities were examined using principal coordinates analysis (PCoA). Robustness of results was tested by recomputing output using alternative dissimilarity measures (Jaccard, thetayc) and ordinations (non-metric multi-dimensional scaling (NMDS)). No significant changes were observed (data not shown).

### Spore preparation and enumeration

Individual *C. difficile* colonies were picked into pre-reduced BHIS media and propagated anaerobically at 37 °C overnight. Spores were cultivated by spread plating overnight BHIS cultures onto BHIS medium and incubating anaerobically at 37 °C for 5 days. Spores were scraped from plates, resuspended in sterile water, and heated to 65 °C for 30 min to kill vegetative cells. Viable spores were enumerated by plating serial dilutions on BHIS plates supplemented with 0.1 % taurocholic acid. Spore preparations were diluted in sterile water to a final concentration of 5 × 10^6^ spores ml^−1^ and maintained at 4 °C until used. To enumerate *C. difficile* spores from ^HMb^mouse feces, fecal pellets were weighed, homogenized in 500 μl sterile water, and incubated at 65 °C for 30 min to kill vegetative cells. Following heat killing, spores were serially diluted and spot plated in duplicate onto selective TCCFA plates containing 0.1 % taurocholic acid.

### Establishment of robust *C. difficile* infection model in ^HMb^mice

To produce a robust infection model in ^HMb^mice, a number of antibiotic pretreatment regimes, known to work well in ^CMb^mice, were tested including the following: cefoperazone (0.5 mg ml^−1^) in sterile drinking water for 10 days [50] and clindamycin (0.25 mg ml^−1^) in drinking water for 7 days [51]. However, only the use of a five-antibiotic cocktail developed by Chen et al. [9] produced reproducible results in ^HMb^mice. ^HMb^Mice were administered kanamycin (0.4 mg ml^−1^), gentamicin (0.035 mg ml^−1^), colistin (850 U ml^−1^), metronidazole (0.215 mg ml^−1^), and vancomycin (0.045 mg ml^−1^) ad libitum in drinking water for 3–5 days followed 24 h later by IP injection of clindamycin (10 mg kg^−1^). Twenty-four hours post IP injection, mice were challenged, via oral gavage, with 5 × 10^5^ spores of either the high toxin producing strain VPI10463 or the clinically relevant NAP1/B1/027 ribotype strain CD3017. Symptoms were similar to those observed in humans: diarrhea, weight loss, and histologic damage.

### CDI recurrence/relapse

^HMb^Mice were administered the five-antibiotic cocktail for 4 days ad libitum in drinking water followed by IP clindamycin injection. Twenty-four hours later, mice were challenged with 5 × 10^4^ CD3017 spores (tenfold lower compared to prior experiments was used to minimize mouse mortality while maintaining CDI symptoms). Following recovery from initial CDI, relapse was triggered by administering a further single dose of clindamycin (10 mg kg^−1^) up to 4 weeks post recovery from initial infection.

### Histopathology

Histological sections of distal colon were scored blindly by an independent researcher utilizing the scoring system outlined in Theriot et al. [50]. Briefly, three criteria: edema, cellular infiltration, and epithelial damage were individually scored from 0–4 (where a score of 0 indicates no pathology) and the values summed to give an overall disease severity score.

## Additional files

Additional file 1:
**Clustering of humanized mouse microbiota based upon principal coordinates analysis (PCoA) of Bray-Curtis dissimilarities.** A distinction along axis 1, accounting for 41.3 % dissimilarity, can be visualized between humanized mouse microbiota founder mice and their offspring. (PNG 120 kb) Additional file 2:
**Differentially abundant OTUs in pre-antibiotic vs post-antibiotic** ^**HMb**^ **mice communities.** Two hundred and eighteen OTUs were significantly affected by antibiotic treatment. One hundred and ninety eight were significantly reduced while 20 OTUs were able to resist the antibiotic insult and increase in abundance. Significant differential abundances of OTUs were analyzed using DESeq2 in R. *p* values were corrected using the Benjamini-Hochberg false discovery rate (FDR) procedure with a corrected alpha value cutoff of 0.01. (PNG 120 kb) Additional file 3:
**Differentially abundant OTUs in pre-antibiotic vs recovered** ^**HMb**^ **mice communities.** The ability of ^HMb^mouse microbiota to return to a “normal” state following recovery from antibiotic insult and CDI was tested by comparing the two groups: pretreatment (no antibiotics, *n* = 38) and recovered (14–17 days post antibiotic cessation and CDI, *n* = 25). Significant differential abundances of OTUs were analyzed using DESeq2 in R. *p* values were corrected using the Benjamini-Hochberg false discovery rate (FDR) procedure with a corrected alpha value cutoff of 0.01. (PDF 260 kb)
