# Supplementary figures and images for: Humanized microbiota mice as a model of recurrent Clostridium difficile disease
Source: Microbiome. 2015 Aug 20;3:35. doi: 10.1186/s40168-015-0097-2 (PMC4546040; doi:10.1186/s40168-015-0097-2)

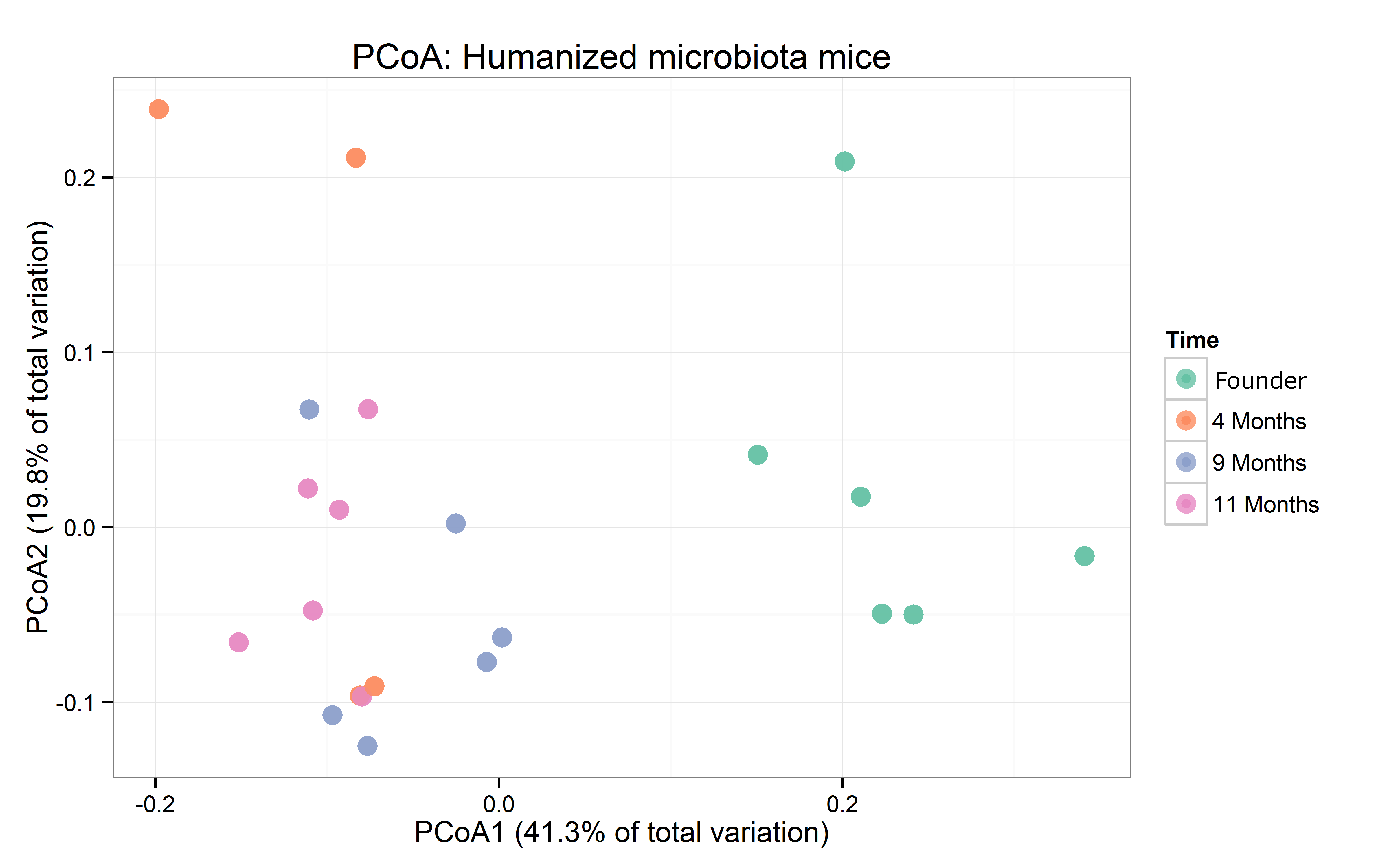

Supplement: Additional file 1: — Clustering of humanized mouse microbiota based upon principal coordinates analysis (PCoA) of Bray-Curtis dissimilarities. A distinction along axis 1, accounting for 41.3 % dissimilarity, can be visualized between humanized mouse microbiota founder mice and their offspring. (PNG 120 kb) [file 40168_2015_97_MOESM1_ESM.png]
