# Supplementary material for: Humanized microbiota mice as a model of recurrent Clostridium difficile disease
Source: Microbiome. 2015 Aug 20;3:35. doi: 10.1186/s40168-015-0097-2 (PMC4546040; doi:10.1186/s40168-015-0097-2)
Supplement: Additional file 2: — Differentially abundant OTUs in pre-antibiotic vs post-antibiotic HMb mice communities. Two hundred and eighteen OTUs were significantly affected by antibiotic treatment. One hundred and ninety eight were significantly reduced while 20 OTUs were able to resist the antibiotic insult and increase in abundance. Significant differential abundances of OTUs were analyzed using DESeq2 in R. p values were corrected using the Benjamini-Hochberg false discovery rate (FDR) procedure with a corrected alpha value cutoff of 0.01. (PNG 120 kb) [file 40168_2015_97_MOESM2_ESM.pdf]

Differentially abundant OTUs in Pre-antibiotic Vs Post-antibiotic HMb mice communities.

Two-hundered and eighteen OTUs were significantly affected by antibiotic treatment. One hundred and ninety-eight were significantly reduced whilst 20 OTUs were able to resist the antibiotic insult and increase in abundance. Significant differential abundances of OTUs were analyzed using DESeq2 in R. P values were corrected using the Benjamini-Hochberg False Discovery Rate (FDR) procedure with a corrected alpha value cutoff of 0.01.

	baseMean	log2FoldChange	Order	Family	Genus
**Otu00040**	421.52	-14.44	Clostridiales	unclassified	unclassified
**Otu00014**	354.76	-13.96	Clostridiales	Lachnospiraceae	unclassified
**Otu00001**	2911.5	-13.42	unclassified	unclassified	unclassified
**Otu00037**	115.94	-12.98	Clostridiales	Lachnospiraceae	unclassified
**Otu00024**	182.48	-12.87	Clostridiales	Lachnospiraceae	unclassified
**Otu00008**	920.69	-12.83	Clostridiales	Lachnospiraceae	unclassified
**Otu00061**	131.8	-12.78	Burkholderiales	Sutterellaceae	Sutterella
**Otu00025**	140.25	-12.77	Clostridiales	Lachnospiraceae	unclassified
**Otu00009**	1875.46	-12.75	Clostridiales	Lachnospiraceae	unclassified
**Otu00015**	200.67	-12.58	Clostridiales	Lachnospiraceae	unclassified
**Otu00063**	122.32	-12.03	Clostridiales	Lachnospiraceae	unclassified
**Otu00080**	93.07	-11.8	Clostridiales	Ruminococcaceae	unclassified
**Otu00120**	66	-11.78	Clostridiales	Lachnospiraceae	unclassified
**Otu00087**	52.26	-11.66	Clostridiales	Lachnospiraceae	unclassified
**Otu00032**	138.52	-11.62	Clostridiales	Lachnospiraceae	unclassified
**Otu00016**	97.12	-11.55	Clostridiales	Lachnospiraceae	unclassified
**Otu00027**	67.19	-11.42	Clostridiales	Lachnospiraceae	unclassified
**Otu00053**	66.66	-11.42	Clostridiales	Lachnospiraceae	Roseburia
**Otu00026**	349.15	-11.38	Clostridiales	Ruminococcaceae	Oscillibacter
**Otu00060**	37.33	-11.37	Clostridiales	Lachnospiraceae	unclassified
**Otu00410**	38.98	-11.33	unclassified	unclassified	unclassified
**Otu00088**	127.2	-11.33	Clostridiales	Lachnospiraceae	unclassified
**Otu00109**	33.65	-11.2	Clostridiales	unclassified	unclassified
**Otu00104**	30.87	-11.08	Clostridiales	Lachnospiraceae	unclassified
**Otu00085**	67.67	-11.05	Clostridiales	Lachnospiraceae	unclassified
**Otu00146**	34.27	-11	Clostridiales	unclassified	unclassified
**Otu00096**	29.85	-10.9	Clostridiales	Ruminococcaceae	unclassified
**Otu00143**	85.92	-10.84	Clostridiales	Lachnospiraceae	unclassified
**Otu00058**	55.32	-10.77	Clostridiales	Ruminococcaceae	Butyricicoccus
**Otu00074**	22.46	-10.55	Clostridiales	Lachnospiraceae	unclassified
**Otu00059**	31.1	-10.47	Clostridiales	Lachnospiraceae	Roseburia
**Otu00006**	42.56	-10.42	Bacteroidales	unclassified	unclassified
**Otu00019**	72.81	-10.38	Clostridiales	Lachnospiraceae	unclassified
**Otu00065**	33.14	-10.36	Clostridiales	Lachnospiraceae	unclassified
**Otu00132**	17.54	-10.31	Clostridiales	Lachnospiraceae	unclassified
**Otu00121**	17.54	-10.3	Clostridiales	Lachnospiraceae	unclassified
**Otu00169**	22.71	-10.21	Clostridiales	Lachnospiraceae	unclassified
**Otu00028**	149.99	-10.14	Clostridiales	Lachnospiraceae	unclassified
**Otu00031**	84.81	-10	Clostridiales	Lachnospiraceae	Clostridium_XlVa
**Otu00097**	20.62	-9.99	Clostridiales	Lachnospiraceae	Clostridium_XlVa
**Otu00099**	24.19	-9.97	Clostridiales	Lachnospiraceae	unclassified
**Otu00241**	13.58	-9.93	Clostridiales	unclassified	unclassified
**Otu00157**	13.1	-9.87	Clostridiales	Lachnospiraceae	unclassified
**Otu00100**	18.88	-9.82	Clostridiales	Lachnospiraceae	unclassified
**Otu00197**	12.91	-9.82	Clostridiales	Lachnospiraceae	unclassified
**Otu00155**	11.39	-9.49	Clostridiales	Lachnospiraceae	unclassified
**Otu00309**	9.89	-9.44	unclassified	unclassified	unclassified
**Otu00105**	9.61	-9.42	Clostridiales	Lachnospiraceae	unclassified
**Otu00117**	12.44	-9.41	Clostridiales	Lachnospiraceae	unclassified
**Otu00177**	10	-9.28	Clostridiales	Lachnospiraceae	unclassified
**Otu00089**	11.15	-9.19	Clostridiales	Lachnospiraceae	unclassified
**Otu00066**	17.45	-9.12	Clostridiales	Lachnospiraceae	unclassified
**Otu00194**	7.62	-9.01	Clostridiales	Lachnospiraceae	unclassified

**Otu00163**	11.18	-8.96	Clostridiales	Lachnospiraceae	unclassified
**Otu00078**	6.75	-8.91	Clostridiales	Ruminococcaceae	unclassified
**Otu00072**	6.73	-8.84	Clostridiales	Lachnospiraceae	unclassified
**Otu00209**	6.62	-8.83	Clostridiales	Lachnospiraceae	unclassified
**Otu00579**	6.14	-8.72	Clostridiales	unclassified	unclassified
**Otu00013**	158.29	-8.65	Clostridiales	Lachnospiraceae	unclassified
**Otu00244**	5.08	-8.56	Bacteroidales	Rikenellaceae	Alistipes
**Otu00114**	5.14	-8.53	Clostridiales	Lachnospiraceae	unclassified
**Otu00098**	23.55	-8.5	Clostridiales	Ruminococcaceae	unclassified
**Otu00103**	5.09	-8.5	Clostridiales	Lachnospiraceae	unclassified
**Otu00128**	4.71	-8.41	Clostridiales	unclassified	unclassified
**Otu00055**	77.88	-8.36	Clostridiales	Ruminococcaceae	unclassified
**Otu00144**	12.8	-8.35	Clostridiales	Ruminococcaceae	unclassified
**Otu00135**	4.41	-8.31	Clostridiales	Lachnospiraceae	unclassified
**Otu00165**	10.02	-8.3	Clostridiales	Lachnospiraceae	unclassified
**Otu00255**	4.32	-8.21	Clostridiales	Lachnospiraceae	unclassified
**Otu00118**	4.5	-7.99	Clostridiales	Lachnospiraceae	unclassified
**Otu00047**	100.31	-7.94	Clostridiales	Lachnospiraceae	unclassified
**Otu00264**	4.42	-7.87	Clostridiales	unclassified	unclassified
**Otu00302**	4.26	-7.82	unclassified	unclassified	unclassified
**Otu01189**	2.92	-7.73	Bacteroidales	Rikenellaceae	Alistipes
**Otu00437**	4.86	-7.67	Clostridiales	Ruminococcaceae	unclassified
**Otu00124**	2.79	-7.66	Coriobacteriales	Coriobacteriaceae	unclassified
**Otu00224**	7.5	-7.58	Clostridiales	Ruminococcaceae	unclassified
**Otu00231**	2.69	-7.57	Clostridiales	Ruminococcaceae	Butyricicoccus
**Otu01865**	2.51	-7.45	Erysipelotrichales	Erysipelotrichaceae	Coprobacillus
**Otu00035**	3.1	-7.42	Clostridiales	Lachnospiraceae	unclassified
**Otu00284**	2.48	-7.39	Clostridiales	Lachnospiraceae	unclassified
**Otu01095**	2.54	-7.36	Clostridiales	Lachnospiraceae	unclassified
**Otu00196**	2.29	-7.31	unclassified	unclassified	unclassified
**Otu00149**	13.47	-7.3	Clostridiales	Lachnospiraceae	unclassified
**Otu00139**	7.64	-7.3	Clostridiales	Lachnospiraceae	unclassified
**Otu00168**	3.52	-7.17	Clostridiales	Ruminococcaceae	unclassified
**Otu00399**	2.72	-7.16	Clostridiales	Lachnospiraceae	unclassified
**Otu00042**	1.98	-7.12	unclassified	unclassified	unclassified
**Otu00179**	3.37	-7.06	Clostridiales	Peptostreptococcaceae	Clostridium_XI
**Otu00290**	2.28	-6.97	Coriobacteriales	Coriobacteriaceae	unclassified
**Otu00376**	2.42	-6.95	Clostridiales	Lachnospiraceae	unclassified
**Otu00101**	66.21	-6.94	Clostridiales	unclassified	unclassified
**Otu00320**	1.63	-6.87	Clostridiales	unclassified	unclassified
**Otu00200**	1.57	-6.85	Clostridiales	Ruminococcaceae	unclassified
**Otu00240**	7.25	-6.83	Clostridiales	Ruminococcaceae	Clostridium_IV
**Otu00233**	3.4	-6.74	Coriobacteriales	Coriobacteriaceae	unclassified
**Otu00663**	1.56	-6.71	Clostridiales	Ruminococcaceae	unclassified
**Otu00012**	598.26	-6.69	Bacteroidales	Bacteroidaceae	Bacteroides
**Otu00485**	1.4	-6.63	Clostridiales	Ruminococcaceae	unclassified
**Otu00248**	4.37	-6.51	Bacteroidales	Rikenellaceae	Alistipes
**Otu00133**	12.75	-6.51	Clostridiales	Ruminococcaceae	unclassified
**Otu00136**	23.75	-6.45	Clostridiales	Lachnospiraceae	unclassified
**Otu00182**	1.18	-6.36	Bacteroidales	unclassified	unclassified
**Otu00263**	1.48	-6.34	Clostridiales	Lachnospiraceae	Dorea
**Otu00093**	16.26	-6.21	Clostridiales	Lachnospiraceae	Clostridium_XlVa
**Otu00160**	6.1	-6.2	Coriobacteriales	Coriobacteriaceae	Enterorhabdus
**Otu00094**	4.08	-6.18	Clostridiales	Ruminococcaceae	Anaerotruncus
**Otu00077**	41.8	-6.17	Clostridiales	Lachnospiraceae	unclassified
**Otu00323**	1.85	-6.11	Clostridiales	Ruminococcaceae	unclassified
**Otu00390**	0.94	-6.07	Clostridiales	Ruminococcaceae	unclassified
**Otu00969**	1.01	-6.04	unclassified	unclassified	unclassified
**Otu00123**	2.86	-6.04	Bacteroidales	Bacteroidaceae	Bacteroides
**Otu00052**	1.72	-5.96	Clostridiales	Lachnospiraceae	unclassified
**Otu00154**	29.19	-5.95	Clostridiales	Ruminococcaceae	unclassified
**Otu00138**	13.98	-5.87	Clostridiales	Lachnospiraceae	Blautia
**Otu00521**	0.71	-5.8	Clostridiales	Lachnospiraceae	Anaerostipes
**Otu00150**	1.02	-5.74	Clostridiales	Lachnospiraceae	unclassified
**Otu00353**	0.71	-5.7	unclassified	unclassified	unclassified
**Otu00279**	1.18	-5.59	Clostridiales	Lachnospiraceae	unclassified

**Otu00331**	1.92	-5.57	Clostridiales	Lachnospiraceae	Clostridium_XlVb
**Otu00112**	0.64	-5.53	Bacteroidales	unclassified	unclassified
**Otu00176**	16.36	-5.5	Erysipelotrichales	Erysipelotrichaceae	unclassified
**Otu00092**	0.76	-5.4	Bacteroidales	Bacteroidaceae	Bacteroides
**Otu00815**	0.59	-5.38	Clostridiales	Ruminococcaceae	unclassified
**Otu00038**	46.16	-5.35	Clostridiales	unclassified	unclassified
**Otu00532**	0.76	-5.32	unclassified	unclassified	unclassified
**Otu00148**	0.51	-5.19	Clostridiales	Lachnospiraceae	unclassified
**Otu00306**	0.57	-5.14	Clostridiales	Lachnospiraceae	unclassified
**Otu00134**	0.49	-5.1	Clostridiales	Lachnospiraceae	unclassified
**Otu00534**	0.45	-5.07	unclassified	unclassified	unclassified
**Otu00319**	0.48	-5.06	Clostridiales	Lachnospiraceae	unclassified
**Otu00129**	4.8	-5.05	Clostridiales	Ruminococcaceae	unclassified
**Otu00164**	0.53	-5.05	Clostridiales	Ruminococcaceae	unclassified
**Otu00170**	0.43	-4.94	Clostridiales	Lachnospiraceae	unclassified
**Otu00108**	21.02	-4.93	Clostridiales	Lachnospiraceae	Clostridium_XlVa
**Otu00018**	264.29	-4.87	Clostridiales	unclassified	unclassified
**Otu02722**	0.38	-4.84	Clostridiales	Ruminococcaceae	unclassified
**Otu00126**	0.42	-4.83	Bacteroidales	Bacteroidaceae	Bacteroides
**Otu00174**	4.03	-4.72	Clostridiales	Lachnospiraceae	unclassified
**Otu00187**	0.37	-4.71	Bacteroidales	Bacteroidaceae	Bacteroides
**Otu00717**	0.9	-4.7	Clostridiales	Lachnospiraceae	unclassified
**Otu00021**	469.71	-4.7	Erysipelotrichales	Erysipelotrichaceae	unclassified
**Otu00005**	4837.22	-4.69	Bacteroidales	Bacteroidaceae	Bacteroides
**Otu00107**	0.51	-4.65	Bacteroidales	Bacteroidaceae	Bacteroides
**Otu00494**	0.36	-4.63	Clostridiales	Lachnospiraceae	unclassified
**Otu00064**	144.01	-4.62	Clostridiales	Ruminococcaceae	unclassified
**Otu00287**	0.59	-4.61	Clostridiales	Ruminococcaceae	unclassified
**Otu00115**	0.88	-4.49	unclassified	unclassified	unclassified
**Otu00543**	0.71	-4.46	Clostridiales	Lachnospiraceae	Clostridium_XlVb
**Otu00286**	0.29	-4.38	Clostridiales	Ruminococcaceae	unclassified
**Otu00217**	0.32	-4.37	Bacteroidales	Bacteroidaceae	Bacteroides
**Otu00501**	0.26	-4.36	Clostridiales	Ruminococcaceae	Ruminococcus
**Otu00039**	69.24	-4.35	Clostridiales	unclassified	unclassified
**Otu01125**	0.27	-4.21	Clostridiales	unclassified	unclassified
**Otu00141**	1.74	-4.2	Selenomonadales	Acidaminococcaceae	Phascolarctobacterium
**Otu00336**	0.25	-4.17	Bacteroidales	unclassified	unclassified
**Otu00215**	0.26	-4.11	unclassified	unclassified	unclassified
**Otu00033**	53.71	-4.09	Clostridiales	Lachnospiraceae	Clostridium_XlVa
**Otu00130**	0.59	-4.08	Bacteroidales	Bacteroidaceae	Bacteroides
**Otu00206**	0.23	-4.02	Bacteroidales	Porphyromonadaceae	Parabacteroides
**Otu00161**	0.22	-3.97	Bacteroidales	Bacteroidaceae	Bacteroides
**Otu00862**	0.2	-3.9	Clostridiales	Ruminococcaceae	Clostridium_IV
**Otu00111**	0.38	-3.89	Bacteroidales	Bacteroidaceae	Bacteroides
**Otu00193**	0.22	-3.86	Clostridiales	Lachnospiraceae	unclassified
**Otu00301**	0.28	-3.85	Clostridiales	Lachnospiraceae	Clostridium_XlVa
**Otu00151**	0.25	-3.75	Bacteroidales	Bacteroidaceae	Bacteroides
**Otu00017**	443.34	-3.74	Bacteroidales	Porphyromonadaceae	Parabacteroides
**Otu00041**	300.64	-3.66	Clostridiales	Eubacteriaceae	Eubacterium
**Otu00057**	73.9	-3.65	Clostridiales	Lachnospiraceae	unclassified
**Otu00137**	0.39	-3.61	Clostridiales	Lachnospiraceae	Dorea
**Otu00183**	0.17	-3.59	Bacteroidales	unclassified	unclassified
**Otu00232**	0.15	-3.54	Clostridiales	Lachnospiraceae	unclassified
**Otu00188**	0.14	-3.44	Bacteroidales	unclassified	unclassified
**Otu00269**	0.17	-3.41	Bacteroidales	unclassified	unclassified
**Otu00073**	47.65	-3.41	Clostridiales	Ruminococcaceae	Clostridium_IV
**Otu00228**	0.25	-3.32	Clostridiales	Ruminococcaceae	unclassified
**Otu00747**	0.47	-3.3	Clostridiales	unclassified	unclassified
**Otu00062**	0.39	-3.29	Bacteroidales	Bacteroidaceae	Bacteroides
**Otu00229**	0.23	-3.2	Clostridiales	unclassified	unclassified
**Otu00022**	1025.65	-3.19	Selenomonadales	Acidaminococcaceae	Phascolarctobacterium
**Otu00210**	0.88	-3.18	Clostridiales	Ruminococcaceae	unclassified
**Otu00156**	28.8	-3.07	unclassified	unclassified	unclassified
**Otu00082**	0.66	-3.03	Bacteroidales	Bacteroidaceae	Bacteroides
**Otu00084**	0.42	-2.95	Bacteroidales	Bacteroidaceae	Bacteroides
**Otu00095**	5.78	-2.88	Clostridiales	Lachnospiraceae	unclassified

**Otu00086**	15.64	-2.79	Clostridiales	Lachnospiraceae	unclassified
**Otu00090**	22.62	-2.7	Bacteroidales	Porphyromonadaceae	unclassified
**Otu00067**	1.04	-2.66	Bacteroidales	Bacteroidaceae	Bacteroides
**Otu00116**	8.11	-2.61	Clostridiales	Lachnospiraceae	unclassified
**Otu00048**	40.56	-2.08	Clostridiales	Lachnospiraceae	Clostridium_XlVa
**Otu00079**	1.41	-2.06	Bacteroidales	Bacteroidaceae	Bacteroides
**Otu00051**	55.2	-2.01	Bacteroidales	Porphyromonadaceae	Butyricimonas
**Otu00036**	358.2	-1.51	Desulfovibrionales	Desulfovibrionaceae	Bilophila
**Otu00029**	4.04	-1.23	Bacteroidales	Bacteroidaceae	Bacteroides
**Otu00235**	11.97	-1.01	Burkholderiales	Oxalobacteraceae	unclassified
**Otu00190**	2.34	-0.99	Alteromonadales	Shewanellaceae	Shewanella
**Otu00204**	4.91	-0.9	Oceanospirillales	Halomonadaceae	Halomonas
**Otu00020**	469.75	-0.82	Bacteroidales	Rikenellaceae	Alistipes
**Otu00004**	1228.11	0.58	Bacteroidales	Porphyromonadaceae	Parabacteroides
**Otu00003**	4110.01	0.75	Bacteroidales	Bacteroidaceae	Bacteroides
**Otu00070**	57.65	0.87	Clostridiales	Ruminococcaceae	Flavonifractor
**Otu00007**	2568.19	1.07	Bacteroidales	Bacteroidaceae	Bacteroides
**Otu00145**	4.98	1.16	Clostridiales	Ruminococcaceae	unclassified
**Otu00081**	33.5	1.53	Clostridiales	unclassified	unclassified
**Otu00056**	185.45	1.94	Clostridiales	Ruminococcaceae	Subdoligranulum
**Otu00106**	0.64	2.83	Bacteroidales	unclassified	unclassified
**Otu00153**	7.85	2.88	Clostridiales	unclassified	unclassified
**Otu00166**	0.22	3.12	Bacteroidales	Bacteroidaceae	Bacteroides
**Otu00023**	794.25	3.28	Burkholderiales	Sutterellaceae	Parasutterella
**Otu00377**	5.21	3.49	Clostridiales	unclassified	unclassified
**Otu00257**	0.32	3.53	unclassified	unclassified	unclassified
**Otu00172**	269.62	3.72	unclassified	unclassified	unclassified
**Otu00152**	0.54	3.83	unclassified	unclassified	unclassified
**Otu00050**	257.7	4.77	Erysipelotrichales	Erysipelotrichaceae	Erysipelotrichaceae_incertae_sedis
**Otu00238**	3.02	5	Clostridiales	unclassified	unclassified
**Otu00049**	258.85	5.95	unclassified	unclassified	unclassified
**Otu00127**	183.71	7.14	unclassified	unclassified	unclassified
**Otu00043**	159.44	8.48	Lactobacillales	Enterococcaceae	Enterococcus
