# Supplementary material for: Humanized microbiota mice as a model of recurrent Clostridium difficile disease
Source: Microbiome. 2015 Aug 20;3:35. doi: 10.1186/s40168-015-0097-2 (PMC4546040; doi:10.1186/s40168-015-0097-2)
Supplement: Additional file 3: — Differentially abundant OTUs in pre-antibiotic vs recovered HMb mice communities. The ability of HMbmouse microbiota to return to a “normal” state following recovery from antibiotic insult and CDI was tested by comparing the two groups: pretreatment (no antibiotics, n = 38) and recovered (14–17 days post antibiotic cessation and CDI, n = 25). Significant differential abundances of OTUs were analyzed using DESeq2 in R. p values were corrected using the Benjamini-Hochberg false discovery rate (FDR) procedure with a corrected alpha value cutoff of 0.01. (PDF 260 kb) [file 40168_2015_97_MOESM3_ESM.pdf]

Differentially abundant OTUs in Pre-antibiotic Vs Recovered ^{HMb}mice communities.

The ability of ^{HMb}mouse microbiota to return to a `normal' state following recovery from antibiotic insult and CDI was tested by comparing the two groups: Pre-treatment (no antibiotics, n=38) and Recovered (14-17 days post antibiotic cessation and CDI, n=25). Significant differential abundances of OTUs were analyzed using DESeq2 in R. P values were corrected using the Benjamini-Hochberg False Discovery Rate (FDR) procedure with a corrected alpha value cutoff of 0.01.

OTU	baseMean	log2FoldChange	Order	Family	Genus
**OTU00080**	71.13	-10.11	Clostridiales	Ruminococcaceae	unclassified
**OTU00109**	27.24	-9.13	Clostridiales	unclassified	unclassified
**OTU00169**	16.50	-7.01	Clostridiales	Lachnospiraceae	unclassified
**OTU00264**	3.58	-6.26	Clostridiales	unclassified	unclassified
**OTU01865**	2.18	-5.59	Erysipelotrichales	Erysipelotrichaceae	Coprobacillus
**OTU00061**	117.92	-5.55	Burkholderiales	Sutterellaceae	Sutterella
**OTU00284**	2.23	-5.46	Clostridiales	Lachnospiraceae	unclassified
**OTU00395**	4.13	-4.5	Clostridiales	Lachnospiraceae	Clostridium_XlVb
**OTU00717**	0.74	-4.25	Clostridiales	Lachnospiraceae	unclassified
**OTU00323**	1.42	-4.23	Clostridiales	Ruminococcaceae	unclassified
**OTU00410**	32.78	-4.02	unclassified	unclassified	unclassified
**OTU00196**	1.91	-3.44	unclassified	unclassified	unclassified
**OTU00319**	0.33	-3.37	Clostridiales	Lachnospiraceae	unclassified
**OTU00165**	9.23	-2.71	Clostridiales	Lachnospiraceae	unclassified
**OTU00141**	1.53	-2.07	Selenomonadales	Acidaminococcaceae	Phascolarctobacterium
**OTU00022**	967.74	-1.95	Selenomonadales	Acidaminococcaceae	Phascolarctobacterium
**OTU00077**	45.64	-1.05	Clostridiales	Lachnospiraceae	unclassified
**OTU00012**	671.38	-0.81	Bacteroidales	Bacteroidaceae	Bacteroides
**OTU00041**	328.46	-0.75	Clostridiales	Eubacteriaceae	Eubacterium
**OTU00044**	37.16	0.43	Clostridiales	Lachnospiraceae	Clostridium_XlVa
**OTU00020**	517.85	0.53	Bacteroidales	Rikenellaceae	Alistipes
**OTU00121**	29.52	0.78	Clostridiales	Lachnospiraceae	unclassified
**OTU00099**	44.09	0.94	Clostridiales	Lachnospiraceae	unclassified
**OTU00066**	32	1.07	Clostridiales	Lachnospiraceae	unclassified
**OTU00016**	168.55	1.1	Clostridiales	Lachnospiraceae	unclassified
**OTU00029**	6.42	1.11	Bacteroidales	Bacteroidaceae	Bacteroides
**OTU00058**	105.54	1.21	Clostridiales	Ruminococcaceae	Butyricicoccus
**OTU00244**	12.47	1.26	Bacteroidales	Rikenellaceae	Alistipes
**OTU00200**	3.99	1.37	Clostridiales	Ruminococcaceae	unclassified
**OTU00128**	12.42	1.71	Clostridiales	unclassified	unclassified
**OTU00062**	1.16	1.72	Bacteroidales	Bacteroidaceae	Bacteroides
**OTU00068**	2.09	1.86	Bacteroidales	Bacteroidaceae	Bacteroides
**OTU00076**	3.14	1.9	Bacteroidales	Bacteroidaceae	Bacteroides
**OTU00353**	2.62	2.1	unclassified	unclassified	unclassified
**OTU00023**	321.55	2.14	Burkholderiales	Sutterellaceae	Parasutterella
**OTU01245**	0.5	2.69	Lactobacillales	Streptococcaceae	Lactococcus
**OTU00346**	0.19	3.22	Bacteroidales	Bacteroidaceae	Bacteroides
**OTU00497**	0.31	3.31	Clostridiales	Clostridiaceae_1	Clostridium_sensu_stricto
**OTU00185**	0.26	3.55	Bacteroidales	Bacteroidaceae	Bacteroides
**OTU00046**	366.77	3.59	Verrucomicrobiales	Verrucomicrobiaceae	Akkermansia
**OTU00127**	16.77	4.32	unclassified	unclassified	unclassified
**OTU00147**	1.85	5.5	Clostridiales	Peptostreptococcaceae	Clostridium_XI
**OTU00147**	1.85	5.5	Clostridiales	Peptostreptococcaceae	Clostridium_XI
